# Supplementary material for: On the Origin and Evolution of Drosophila New Genes during Spermatogenesis
Source: Genes (Basel). 2021 Nov 15;12(11):1796. doi: 10.3390/genes12111796 (PMC8621406; doi:10.3390/genes12111796)
Supplement: Supplementary file 1 [file genes-12-01796-s001.zip › genes-1397974-supplementary figure.pdf]

Supplementary figures

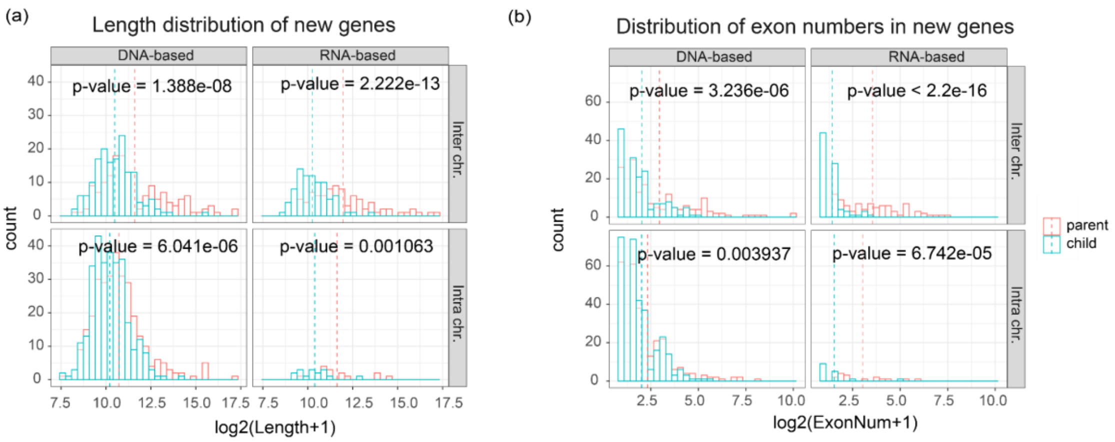

Figure S1. Distribution of gene length and exon numbers of new genes. **(a)** gene length distribution of new genes. **(b)** Distribution of exon numbers in new genes. Statistical tests were using Wilcoxon rank sum test in r.

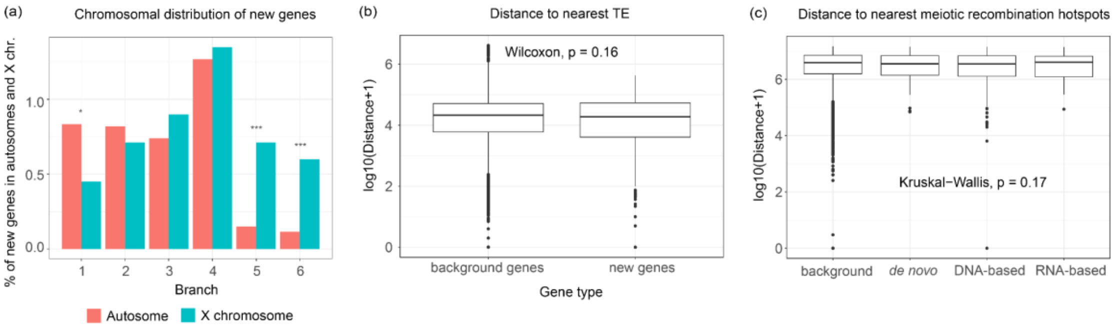

Figure S2. Chromosome location of the newly born genes. **(a)** Chromosome distribution of new genes. Statistic tests were done using Fisher's exact test (p = 0.01972 for branch1, 2.492e-06 for branch 5 and 7.498e-06 for branch 6). **(b)** Distance between genes and nearest TE. (c) Distance between genes and nearest meiotic recombination hotspots. Meiotic recombination hotspots regions were from Chan's paper[27]. Background genes means all other genes besides the new genes.

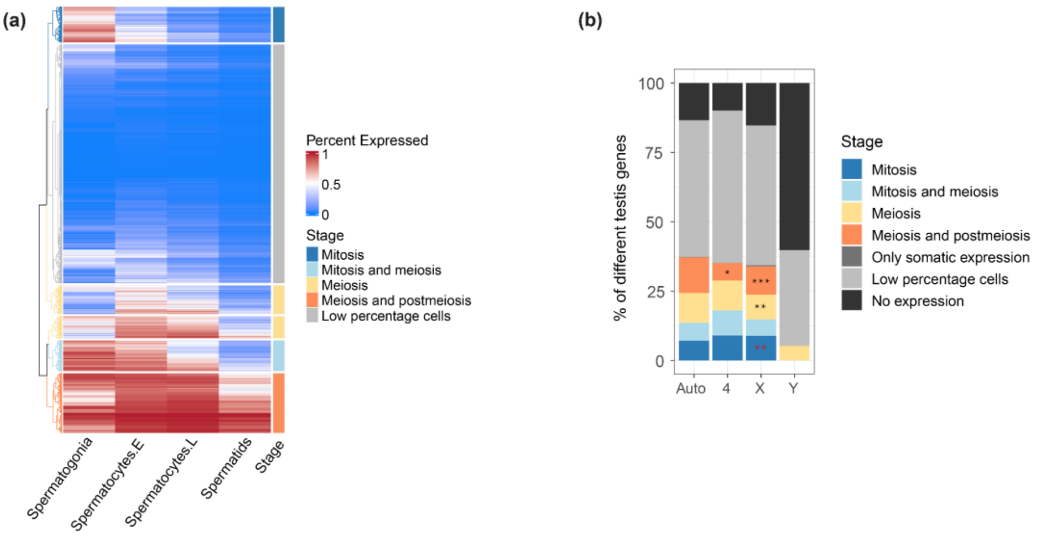

Figure S3. Comparing chromosomal distribution of genes across stages of spermatogenesis based on the percentage of expressed cells. (a) Heatmap of testis-expressing genes in the four cell types based on the percentage of cells expressing the gene in the cell type. 'Low percentage cells' mean this cluster of genes are only expressed in a small proportion of the

cells in all four cell types. (b) The distribution of different types of genes on autosomes, dot chromosome, X and Y chromosomes based on fig. S2a. As postmeiosis stage only have few expressed genes, we combined the postmeiosis stage(red) and ‘meiosis and postmeiosis’ stage(orange) together to perform Fisher’s test and plot the stars between the orange and red bars. The black asterisks means significant underrepresentation when being compared to autosomes, while the red asterisks means significant enrichment.

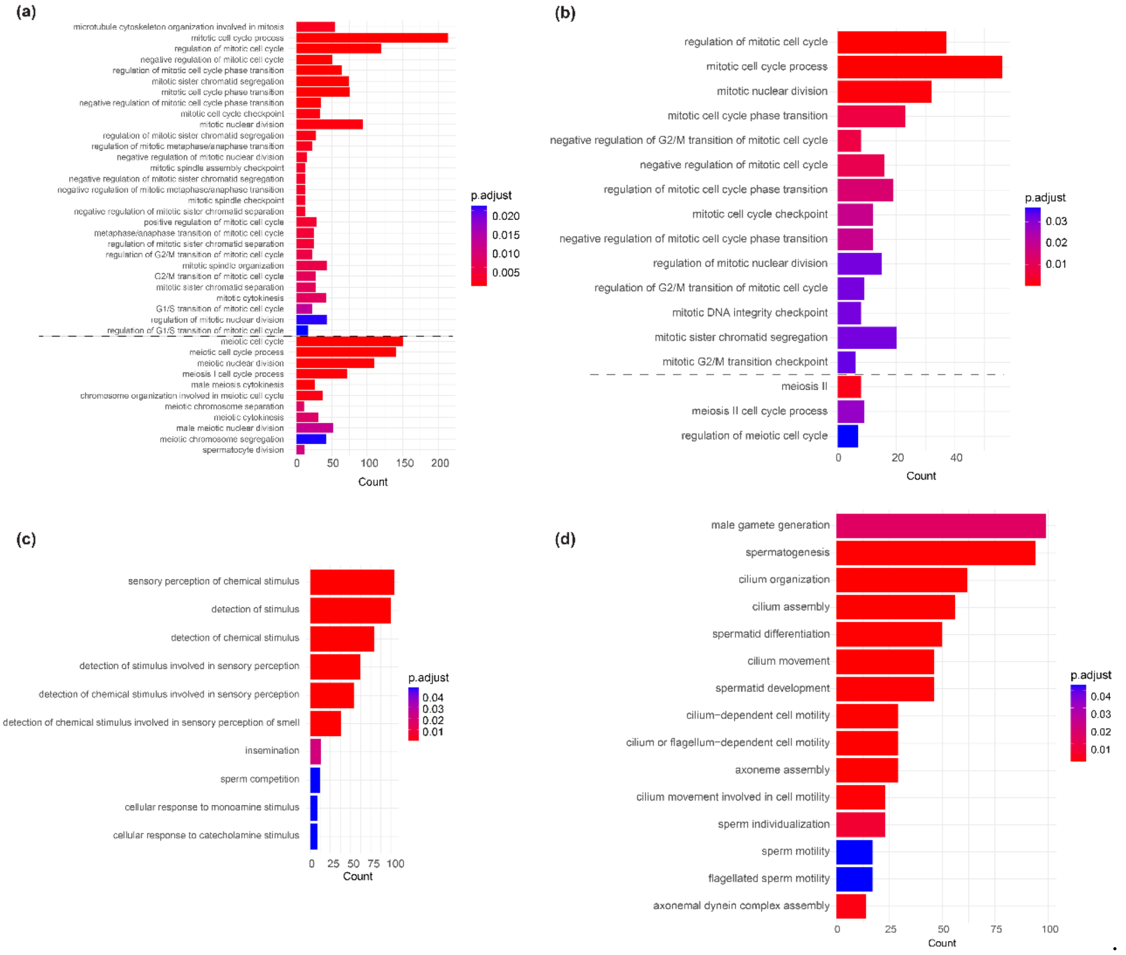

Figure S4. The GO annotation of genes in different stages. (a) GO enrichment of genes enriched in mitosis stage; **(b)** GO enrichment of genes enriched in ‘mitosis and meiosis’ stage. meiosis and mitosis related functions were divided by the dash line; **(c)** GO enrichment of genes enriched in meiosis stage; **(d)** GO enrichment of genes enriched in ‘meiosis and postmeiosis’ stage.

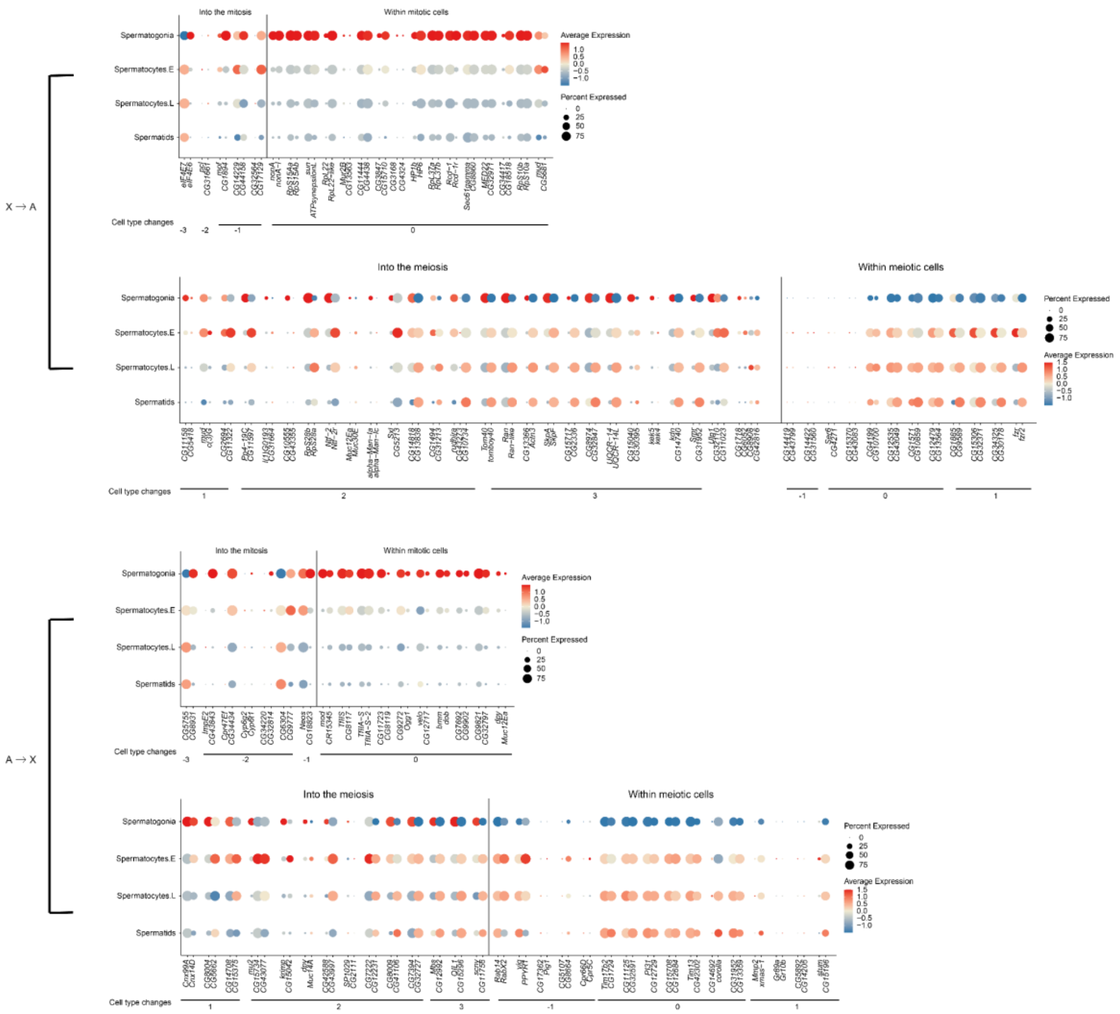

Figure S5. Comparing the expression level between parental and new genes of 'X to A' and 'A to X'. The size of bubbles is scaled to the percentage of cells expressing the gene in the respective cell type. The color is scaled to the normalized expression level, i.e., the log2(CPM+1) value per gene within the respective testis cell type. The pairs were divided into 'X to A' and 'A to X' based on the duplicated orientation. The definitions of 'into the mitosis', 'into meiosis', 'within mitotic cells' and 'within meiotic cells' are consistent with fig3. The numbers in the bottom represent the extent and direction of the cell type changes between parental gene and the new gene. For two genes that are close to each other, the first gene is the parental gene, and the other is the new gene.
